# Photonic Crystal Fiber-Based Surface Plasmon Resonance Sensor with Selective Analyte Channels and Graphene-Silver Deposited Core

**DOI:** 10.3390/s150511499

**Published:** 2015-05-19

**Authors:** Ahmmed A. Rifat, G. Amouzad Mahdiraji, Desmond M. Chow, Yu Gang Shee, Rajib Ahmed, Faisal Rafiq Mahamd Adikan

**Affiliations:** 1 Integrated Lightwave Research Group, Dept. of Electrical Engineering, Faculty of Engineering, University of Malaya, Kuala Lumpur-50603, Malaysia; E-Mails: desmondmcchow@gmail.com (D.M.C.); sheeyg@um.edu.my (Y.G.S.); rafiq@um.edu.my (F.R.M.A.); 2 Institut für Hochfrequenztechnik, Technische Universität Berlin, Einsteinufer 25, 10587 Berlin, Germany; E-Mail: rajib.ahmed.apece@gmail.com

**Keywords:** photonic crystal fiber, surface plasmon resonance, optical fiber sensors, optical sensing and sensors

## Abstract

We propose a surface plasmon resonance (SPR) sensor based on photonic crystal fiber (PCF) with selectively filled analyte channels. Silver is used as the plasmonic material to accurately detect the analytes and is coated with a thin graphene layer to prevent oxidation. The liquid-filled cores are placed near to the metallic channel for easy excitation of free electrons to produce surface plasmon waves (SPWs). Surface plasmons along the metal surface are excited with a leaky Gaussian-like core guided mode. Numerical investigations of the fiber’s properties and sensing performance are performed using the finite element method (FEM). The proposed sensor shows maximum amplitude sensitivity of 418 Refractive Index Units (RIU^−1^) with resolution as high as 2.4 × 10^−5^ RIU. Using the wavelength interrogation method, a maximum refractive index (RI) sensitivity of 3000 nm/RIU in the sensing range of 1.46–1.49 is achieved. The proposed sensor is suitable for detecting various high RI chemicals, biochemical and organic chemical analytes. Additionally, the effects of fiber structural parameters on the properties of plasmonic excitation are investigated and optimized for sensing performance as well as reducing the sensor’s footprint.

## 1. Introduction

In the last few decades, photonic biosensors had shown remarkable development in various applications such as medical diagnostics, bio-chemical detection and organic chemical detection [1,2,3,4,5]. To date, several sensing techniques are available such as micro-ring resonator sensors [6], surface plasmon resonance (SPR) sensors [1,3], *etc.* Surface plasmons (SPs) are the collective oscillations of free electrons that propagate along a metal-dielectric interface by satisfying certain resonance conditions [3,7]. The first SPR experiment for bio-sensing and gas detection was reported by Liedberg *et al.* in 1983 [8], since then, SPR has received much attention due to its extremely sensitive performance. In the conventional Kretschmann SPR configuration, a coupling prism with a thin metal-deposited base is used, and in the presence of incident light at a specific angle, SPs will be excited on the metal-dielectric interface. However, the conventional SPR configuration is bulky and not suitable for remote sensing, which limits its large scale fabrication for real time applications [9].

To overcome this limitation, the feasibility of fiber-based SPR sensors in sensing applications is explored. By harnessing the advantages of photonic-crystal fibers (PCFs) such as small-size and design flexibility, it is possible to control the evanescent field, propagate light in single mode as well as launching light at naught incidence angle into the core to excite SPs. Besides, increased sensitivity and enhanced sensing range are possible by optimizing the PCF structural parameters. Phase matching between the core-guided mode and the surface plasmon polaritons (SPPs) mode could be achieved by carefully designing the fiber structure. In general, a sharp loss peak appears at the phase matching wavelength that enables the sample analyte to be detected [10]. The sensing performance depends highly on the choice of plasmonic materials, and in most cases, gold and silver are used. Gold is chemically stable and shows larger resonance peak shifts as compared with the other active plasmonic materials. However, due to the large absorption coefficient, the resonance band of gold is broader, which reduces the sensing accuracy. The other problems associated with gold including the formation of islands in thin gold layers, band to band transitions and surface roughness due to thermal evaporation [11,12]. On the contrary, silver shows a sharper resonance peak as compared with the other plasmonic materials, however silver is not chemically stable and could be oxidized easily [13]. Silver film with a graphene coating has been shown to solve the problem of oxidation since graphene is impermeable to gas molecules as small as helium and therefore inhibits the passage of oxygen [14]. Furthermore, graphene coatings applied on the metal surface can improve the sensing performance due to their high surface to volume ratio. Besides, they increase the absorption of analyte molecules owing to the π-π stacking and possess superior plasmonic properties that are suitable for sensing [15,16]. The fiber-based plasmonic phenomenon realized by replacing the metal film with graphene was first reported by Kim *et al.* [2], where a thin graphene layer was created by using a thermal chemical vapor deposition (TCVD) technique. Sensing performance improvement of SPR sensors with graphene coatings on top of Au/Ag compared with the corresponding SPR sensors without the graphene coating are also reported [9,17]. Recently, Shuai *et al.* reported the use of gold as an active plasmonic material and the selective infiltration of the PCF core with a high refractive index (RI) analyte to introduce the coexistence of positive and negative RI effects [18]. On the other hand, different fiber structures with enhanced evanescent fields in fiber-based SPR sensors were reported. These include elliptical air-hole PCF sensors with four separated microfluidic slots [1]; multi-hole fiber SPR sensors where all the holes are coated with gold and titanium dioxide [19]; selective six metal oxide coating and liquid infiltration inside the six-air-hole with four different sizes of capillary holes in a polymer PCF [12]; and a seven-air-hole liquid infiltration with six metal oxide coating [20]. However, the above mentioned fiber-based SPR sensors are difficult to fabricate due to both their complex fiber structure and the existence of several metallic channels.

In this paper, we propose a simple liquid core PCF plasmonic sensor with selectively filled analyte channels. A silver layer with a graphene coating are used to improve the sensing performance. Our proposed sensor shows higher amplitude sensitivity as compared with devices reported in [19,21] and higher wavelength interrogation sensitivity than sensors reported in [12,22,23]. The metallic channel, liquid cores, and unique air holes arrangement of the proposed sensor introduce a larger evanescent field that results in stronger coupling between the core-guided mode and the SPP mode.

## 2. Methodology

### 2.1. Structural Design and Numerical Analysis

**Figure 1:**
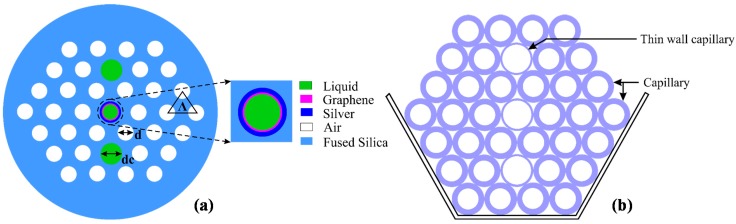
(**a**) Cross-section of the proposed sensor; (**b**) Cross-section of the stacked preform.

Figure 1a shows the cross-section of the proposed fiber sensor. Two identical cores are filled with a high RI liquid (analyte) and form a straight line with the center metallic channel. The proposed structure couples core-guided mode and plasmonic mode at the interface of silver-graphene layer and the analyte. The air-holes are distributed in a triangular lattice where the distance between adjacent air-holes (lattice constant, pitch) is Λ = 1.90 μm and the air-hole diameter is d = 0.5 Λ. The analyte core diameter and the metallic channel diameter are similar, d_1_ = d_c_ = 0.8 Λ. The liquid-filled hole diameter is larger than the other air-holes to simplify the flow of the analyte through them. The silica (SiO_2_) RI profile is approximated by the Sellmeier equation [4]. A graphene layer on top of a thin silver layer is used at the inner wall of the fiber central core. The silver and graphene thicknesses are set as t_ag_ = 35 nm and t_g_ = 3 nm, respectively. The RI of silver is adopted from [24] and the complex RI of the graphene is determined from the equation: n_g_ = 3 + iC_1_λ/3, where λ is the vacuum wavelength in µm and the constant C_1_ ≈ 5.446 μm^−1^. To analyze the guiding properties of the proposed sensor, the finite element method (FEM) is used by considering perfectly matched layer (PML) boundary conditions as a radiation absorber. By taking advantage of the symmetrical structure, we compute only a quarter of the proposed structure to reduce the computation time. Perfect electric conductor (PEC) and perfect magnetic conductor (PMC) boundary conditions are used at the horizontal and vertical outer boundaries, respectively.

### 2.2. Realization of the Proposed Sensor

Fabrication of the proposed fiber structure is possible using the standard stack-and-draw method [25]. The two larger cores and the central metallic channel can be realized by introducing thinner wall capillaries compared to the other capillaries during the stacking process, as illustrated in Figure 1b. The deposition of metal layers on the inner surface of the air-holes in the proposed structure is possible by applying the high pressure chemical vapor deposition (CVD) technique, thermal evaporation, sputtering technique, electroless plating technique or wet-chemistry technique [26,27]. Deposition of graphene layer in the capillary would also be possible by using the CVD method. Ismach *et al.* [28] realized a graphene layer on a dielectric surface using the CVD method. Deposition of graphene on microfibers has also been reported by Wu *et al.* [29]. Besides, growing graphene on silver had been demonstrated by Brain *et al.* [30]. The liquid selective infiltration of single-hole [31] and multi-holes [32] in PCF are also reported by using two-photon direct laser writing and a direct manual gluing method. The proposed sensor can be calibrated with a liquid with known RI and using the flow cell analyte containing process. Since a very short length of optical fiber is required for SPR sensors, the incretion and exchange of analyte inside the fiber holes are practically feasible. As proposed by Wu *et al.* [33], this can be realized by using two short pieces of C-shape fibers placed on the two sides of the PCF SPR sensor. Based on these developed methods, the proposed sensor in this paper has the potential to be mass-fabricated.

## 3. Results and Discussions

### 3.1. Performance Analysis with Respect to Analyte RI

**Figure 2:**
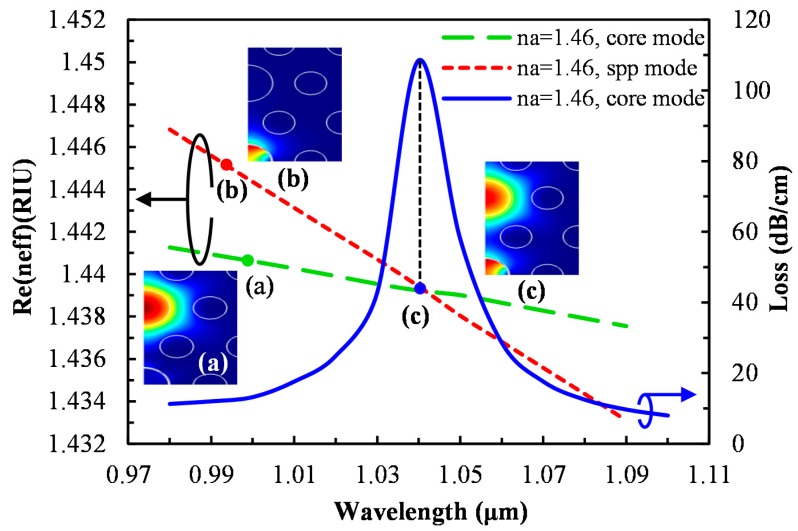
Dispersion relation of the core-guided mode (green), plasmonic mode (red) and the loss spectrum (blue); inset (**a**,**c**) show the electric field of the core-guided mode and inset (**b**) shows the electric field of the plasmonic mode.

The triangular lattice arrangement and the two identical cores perpendicular to the metal-coated channel ensure the polarization-independent propagation characteristics. The fundamental core-guided mode, SPP mode and the resonant spectrum for an analyte with RI, n_a_ = 1.46, are shown in Figure 2.

The real part of effective refractive index (n_eff_) of the core-guided mode and the SPP mode are represented by the green and red dash lines respectively. By using the imaginary part of n_eff_, the propagation loss is determined by the following equation [1]; α = 40π·Im(n_eff_)/(ln(10)λ) ≈ 8.686 × k_0_·Im[n_eff_] dB/m, where k_0_ = 2π/λ is the wave number in free space and λ is the wavelength in μm. A sharp loss peak is found at the resonant wavelength, 1040 nm, where the core-guided fundamental mode and the SPP mode intersect. This indicates the maximum power is transferred from the core-guided fundamental mode to the SPP mode. In Figure 2, for the core-guided fundamental mode (inset (a)), light is well confined in the liquid core, whereas for the SPP mode outside the resonant wavelength (inset (b)), light exists at the metal surface.

**Figure 3:**
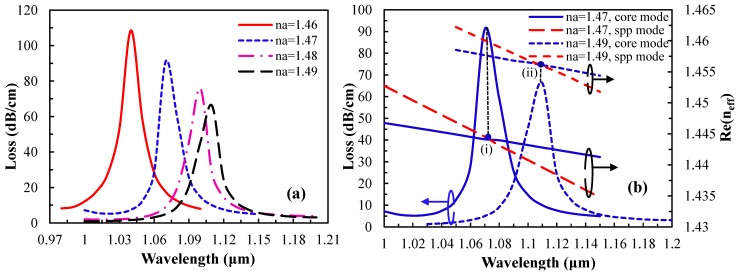
(**a**) Loss spectrum of the fundamental mode by increasing analyte RI, n_a_, from 1.46 to 1.49; (**b**) dispersion relation of the core-guided mode for n_a_ = 1.47 (solid lines) and n_a_ = 1.49 (dashed lines). Red and blue lines indicate SPP mode and the fundamental core-guided mode respectively. Point (I) and (II) are the phase matching points for analyte n_a_ = 1.47 and 1.49.

As for phase matching (inset (c)), fundamental core-mode and SPP mode are coupled with a loss peak at 1040 nm. The proposed sensor is very sensitive in response to the RI of analyte, and a small change in analyte RI leads to large shift in the loss peak. The peak wavelength shift results obtained by varying the analyte RI from 1.46 to 1.49 are shown in Figure 3a.

The real part of n_eff_ of plasmonic mode depends strongly on the vicinity layer of the analyte RI. Due to the small change of analyte RI, the real part of the n_eff_ of SPP mode changes, which causes the change of the phase matching wavelength between the core guided mode and the SPP mode. Mathematically, at the phase matching wavelength, n_eff_ of core guided mode and the SPP mode are approximately the same [34]. In Figure 3a, with the increase of analyte RI, the resonance spectrum shifts toward the longer wavelength and the amplitude of the loss spectrum decreases gradually, and the fundamental mode field confinement also increases. The range of resonant wavelength shift is from 1040 to 1070 nm for the change of analyte RI from 1.46 to 1.47. As such, the positive RI sensitivity is 3000 nm/RIU, which is comparable to results reported in [12,19] using our simpler sensor structure. The sensitivity is determined by following the equation in [1]; Sensitivity, S_λ_(λ) = ∆λ_peak_/∆n_a_, where ∆λ_peak_ is the resonant peak shift and ∆n_a_ is the analyte RI variation. The proposed sensor shows the sensitivity of 3000, 2900 and 1100 nm/RIU for analyte RI variation range of 1.46–1.47, 1.47–1.48 and 1.48–1.49, respectively.

Generally, PCF SPR sensors show high propagation loss, which limits the sensor’s length to generate a measurable signal [34]. The propagation loss of the core guided fundamental mode is a function of analyte RI and the wavelength which is defined as α(λ, n_a_). By considering P_0_ as the input power launched into the fiber, the detected power after propagating through the sensor of length L is P(L, λ, n_a_) = P_0_exp(−α(λ, n_a_)L). By considering a small change of analyte RI dn_a_, the relative sensitivity is defined as S(λ) = [P(L, λ, n_a_ + dn_a_) − P(L, λ, n_a_)]/P(L, λ, n_a_)/dn_a_. Sensor’s length L could be optimized through the measurement of modal transmission loss. A reasonable choice for a sensor length is L = 1/α(λ, n_a_), leading to a simple definition of sensitivity for a small change in analyte RI [1]:

(1) $$S_{A}(\text{λ})[RIU^{-1}]=-\frac{1}{\text{α}(\text{λ},n_{a})}\frac{\partial \text{α}(\text{λ},n_{a})}{\partial n_{a}}$$

**Figure 4:**
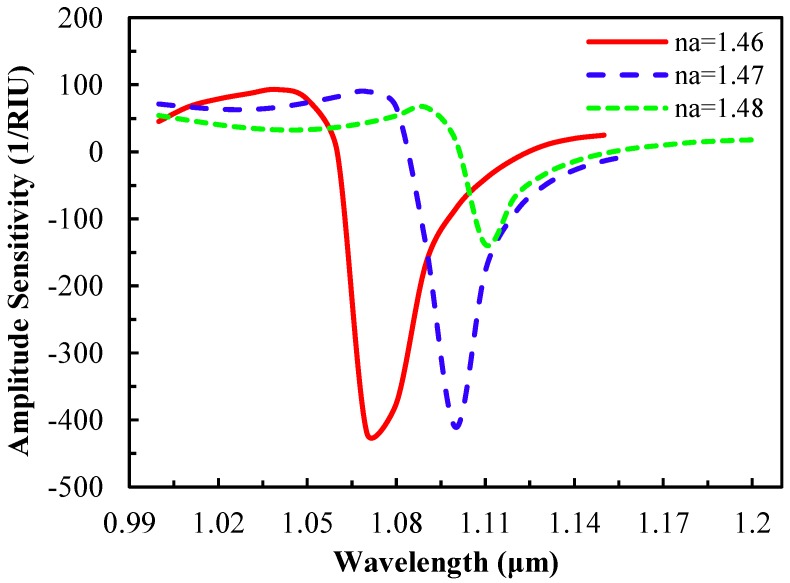
Amplitude sensitivity as a function of wavelength with the variation of analyte RI.

In Figure 3b, it is clear that with the increase of analyte RI, the phase matching wavelength changes. Therefore, at the phase matching resonant wavelength, an unknown sample (analyte) could be detected [1]. As the analyte RI is 1.47, the resonant wavelength is 1070 nm and the loss is 91 dB/cm whereas for n_a_ = 1.49, the resonant wavelength is 1110 nm and the loss is 66 dB/cm. This indicates that with the increase of analyte RI, energy transfer from the core-guided mode to the SPP mode is reduced, and at the same time, the resonance spectrum broadens. In addition, by varying the analyte RI, the amplitude of loss peak changes as seen in Figure 4 (amplitude sensitivity).

The amplitude sensitivity decreases gradually with the increase of analyte RI due to the core-cladding RI contrast. The maximum amplitude sensitivity is 418 RIU^−1^ at 1070 nm wavelength for an analyte of n_a_ = 1.46 which is comparable to [19,21]. With this sensitivity, the resolution is 2.4 × 10^−5^ RIU, assuming that the proposed sensor is able to detect a minimum 1% change of the transmitted light intensity. Amplitude sensitivities of 410 RIU^−1^ and 138 RIU^−1^ are achieved at 1099 nm and 1110 nm for analyte RIs of 1.47 and 1.48, respectively. From the wavelength interrogation and amplitude sensitivity, the proposed sensor can detect high RI liquids in the form of chemical, biochemical and organic chemical analytes.

### 3.2. Performance Analysis and Optimization

**Figure 5:**
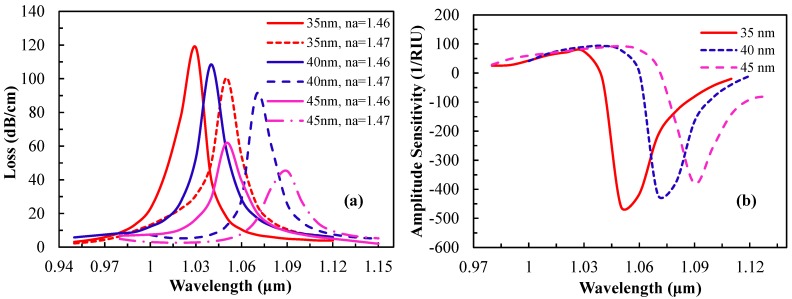
(**a**) Loss spectrum and (**b**) amplitude sensitivity *vs.* wavelength by varying silver thickness, setting analyte RI at n_a_ = 1.46.

The structural parameters of the fiber have a huge influence on the sensing performance. The thickness of the active plasmonic material is important as it has significant effect on the surface plasmon wave excitation. To observe the effect of silver layer thickness on the sensor performance, the silver layer thickness was varied for analyte RIs of 1.46 and 1.47 while the other parameters remain constant. The loss spectrum and the amplitude sensitivity due to the change in silver layer thickness are shown in Figure 5. From Figure 5a, the amplitude of the loss spectrum decreases gradually and shifts toward the longer wavelength with the increase of silver layer thickness from 35 nm to 45 nm. This is due to the higher damping loss for a thicker silver layer [13].

According to Figure 5a, as the silver layer thickness changes from 35 to 45 nm, the resonance peak shifts toward a longer wavelength and the confinement loss gradually decreases. This indicates that due to the increase of silver thickness, light penetration through the cladding decreases. The resonance peak shifts are 20, 30 and 40 nm for silver thicknesses of 35, 40 and 45 nm, respectively. Figure 5b shows the same scenario, where the sensitivity decreases gradually due to the increase of silver thickness. The maximum amplitude sensitivities achieved are 458, 418 and 380 RIU^−1^ for silver thicknesses of 35, 40 and 45 nm, respectively, at an analyte RI of 1.46. This indicates the inverse relation between the sensitivity and the silver layer thickness. The increase of silver layer thickness leads to less penetration of the core mode electric field into the silver layer, resulting in weak coupling with the surface plasmon modes and subsequently affecting the sensitivity. The presence of evanescent fields in the silver layer decreases due to the larger thickness. The amplitude sensitivity of 458, 418 and 380 RIU^−1^ gives sensor resolutions of 2.2 × 10^−5^, 2.4 × 10^−5^ and 2.63 × 10^−5^, respectively, by assuming 1% minimum detectable change in the transmitted light intensity. The thickness of the silver layer is optimized at 40 nm for the study of the other parameters. This typical mechanism could be useful for the studies of nanoparticle concentrations on the metal surface of a sensor [34].

**Figure 6:**
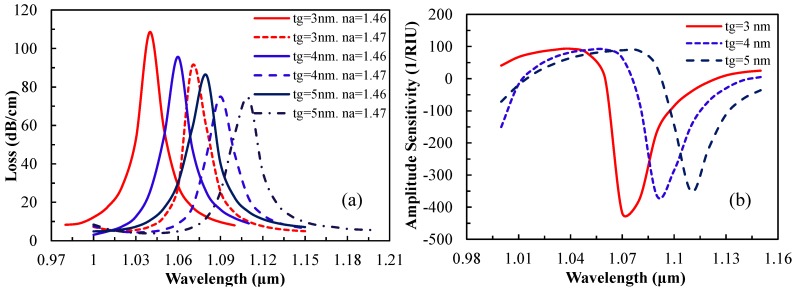
(**a**) Loss spectrum and (**b**) amplitude sensitivity as a function of wavelength by varying graphene layer thickness (analyte n_a_ = 1.46 and silver layer thickness t_ag_ = 40 nm).

Besides the silver layer thickness, the effects of the graphene layer thickness on the loss spectrum and amplitude sensitivity were studied and the related graphs are shown in Figure 6. In Figure 6a, as thickness of the graphene layer increases (3 nm to 5 nm), light confinement in the core improves, which causes less penetration of the core mode electric field into the cladding region. In Figure 6b, as t_g_ increases, the amplitude sensitivity decreases gradually such that 418, 364 and 351 RIU^−1^ is obtained for t_g_ of 3, 4 and 5 nm, respectively. This amplitude sensitivity gives a sensor resolution of 2.4 × 10^−5^, 2.75 × 10^−5^ and 2.85 × 10^−5^ RIU, respectively. The presence of evanescent fields on the metal surface decreases due to the increase in t_g_ which is in agreement with [9]. For SPR sensing, the graphene layer coating on a plasmonic material (silver) shows better sensing performance as compared with a silver layer without a graphene coating. The performance of the sensor improves by 18% as compared to the silver on a bimetallic susbtrate due to the lower damping loss in graphene [9].

**Figure 7:**
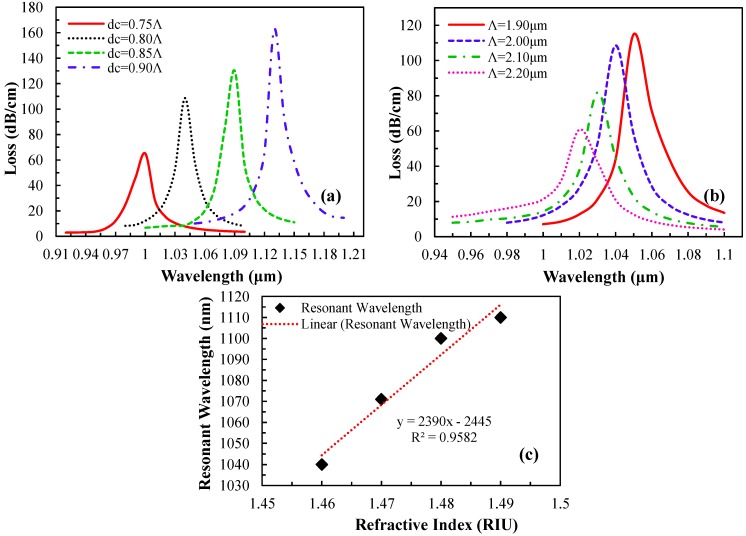
Loss spectrum *vs.* wavelength with the variation of (**a**) metallic core diameter d_c_, (**b**) pitch size Λ (analyte RI, n_a_ = 1.46) and (**c**) linear fitting of the fundamental mode resonant wavelength *vs.* analyte RI.

In addition, the diameter of the metallic core has a significant effect on the surface plasmonic waves. The loss spectrum obtained by changing the metallic core diameters d_c_ while retaining the analyte RI n_a_ = 1.46 is shown in Figure 7a. As d_c_ increases from 0.75Λ to 0.90Λ, the resonant peak shifts toward a longer wavelength and the amplitude of the resonance peak increases gradually, which indicates stronger coupling between the core-guided fundamental mode and SPP modes. In addition, the effect of lattice constant Λ on the sensing performance is shown in Figure 7b. By increasing Λ, the amplitude of the loss spectrum decreases whereas the resonant peak moves toward the shorter wavelength. Unlike increasing d_c_, where the coupling strength between fundamental mode and SPP mode improves, enlarging Λ causes the coupling strength to reduce. Therefore, d_c_ and Λ should be optimized simultaneously to achieve the optimum sensing performance, which have been found as d_c_ = 0.80Λ and Λ = 1.90 μm, respectively.

Figure 7c shows the linear line fitting of the resonant wavelength with respect to the analyte RI. The regression equation is *y* (nm) = 2390*x* − 2445 for 1.46 ≤ *x* ≤ 1.49, where y is the resonant wavelength of the analyte in nm and *x* is the analyte RI. From Figure 7c, the average sensitivity of the proposed sensor is 2390 nm·RIU^−1^ and *R*^2^ value is 0.9582, indicating good fitting of the sensor response. The obtained result is comparable with the results reported in [35]. Owing to the high sensitivity and linearity, the sensor could be implemented as a standardized sensor for high RI analyte detection.

## 4. Conclusions

In this work, we numerically investigate a PCF plasmonic sensor with a graphene-silver deposited layer by full-vectorial FEM. The graphene layer is used to inhibit oxidation of the active plasmonic material, silver. The metallic channel hole and the fiber cores are infiltrated with the sample, a high RI liquid analyte. As the liquid-filled cores satisfy the optimum RI and dispersion relation simultaneously, the SPR sensing performance is increased significantly. Amplitude sensitivity as high as 418 RIU^−1^ has been demonstrated, which gives a sensor resolution of 2.4 × 10^−5^ RIU, assuming that the sensor is able to detect a minimum 1% change of the transmitted intensity. The proposed senor shows a maximum RI sensitivity of 3000 nm/RIU with sensor resolution of 3.33 × 10^−5^ RIU, whereas the average RI sensitivity is 2390 nm/RIU in the sensing range of 1.46 to 1.49.
